# Dietary wheat amylase trypsin inhibitors promote features of murine non-alcoholic fatty liver disease

**DOI:** 10.1038/s41598-019-53323-x

**Published:** 2019-11-25

**Authors:** Muhammad Ashfaq-Khan, Misbah Aslam, Muhammad Asif Qureshi, Marcel Sascha Senkowski, Shih Yen-Weng, Susanne Strand, Yong Ook Kim, Geethanjali Pickert, Jörn M. Schattenberg, Detlef Schuppan

**Affiliations:** 1 Institute of Translational Immunology and Research Center for Immune Therapy, University Medical Center, Johannes Gutenberg University Mainz, Mainz, Germany; 2 Department of Medicine I, University Medical Center, Johannes Gutenberg University, Mainz, Germany; 3 Division of Gastroenterology, Beth Israel Deaconess Medical Center, Harvard Medical School, Boston, MA 02215, USA

**Keywords:** Hepatology, Non-alcoholic fatty liver disease

## Abstract

We previously demonstrated that a common dietary protein component, wheat amylase trypsin inhibitors (ATI), stimulate intestinal macrophages and dendritic cells via toll like receptor 4. Activation of these intestinal myeloid cells elicits an inflammatory signal that is propagated to mesenteric lymph nodes, and that can facilitate extraintestinal inflammation. Mice were fed a well-defined high fat diet, with (HFD/ATI) or without (HFD) nutritionally irrelevant amounts of ATI. Mice on HFD/ATI developed only mild signs of intestinal inflammation and myeloid cell activation but displayed significantly higher serum triglycerides and transaminases compared to mice on HFD alone. Moreover, they showed increased visceral and liver fat, and a higher insulin resistance. ATI feeding promoted liver and adipose tissue inflammation, with M1-type macrophage polarization and infiltration, and enhanced liver fibrogenesis. Gluten, the major protein component of wheat, did not induce these pathologies. Therefore, wheat ATI ingestion in minute quantities comparable to human daily wheat consumption exacerbated features of the metabolic syndrome and non-alcoholic steatohepatitis, despite its irrelevant caloric value.

## Introduction

Non-alcoholic fatty liver disease (NAFLD) is becoming a leading cause of liver related morbidity and mortality in most countries of the world^1–3^. NAFLD is considered the hepatic manifestation of the metabolic syndrome, where obesity, insulin resistance (IR), and type 2 diabetes (T2D) are well-established risk factors that contribute to its progression. NAFLD represents a spectrum of liver diseases that includes simple steatosis, steatosis plus inflammation, hepatocellular ballooning degeneration (NASH), and progression to fibrosis and ultimately cirrhosis, in the absence of excessive alcohol consumption^4–7^. While cardiovascular problems and other complications of the metabolic syndrome prevail in early stages of NAFLD, advanced fibrosis and cirrhosis largely determine the outcome of liver related endpoints^8–10^.

The initiating events in NAFLD are usually dependent on the development of obesity and IR at the level of the adipose tissue and the liver^11^. In addition to these established co-factors, the intestine is emerging as a new site for metabolic and especially immunologic cues that affect whole-body inflammation and metabolism. In line with this, there are multiple independent modifiers of disease risk, including the intestinal microbiota and their metabolites^12–14^ that shape intestinal as well as peripheral immune responses^15^.

Thus, the gut–liver axis has emerged as an important modulator of liver diseases, especially NASH. The liver receives 70% of blood from the gastrointestinal tract^16^ which apart from nutrient resorption also acts as a first line of defense or tolerance towards gut-derived antigens^17,18^. As the main second organ receiving signals from the gut, the liver senses pathogen associated molecular patterns (PAMPs), such as bacterial endotoxins which can reach the portal circulation in case of increased intestinal permeability, thereby promoting necro-inflammation in alcoholic and non-alcoholic steatohepatitis^19^. Furthermore, damage-associated molecular patterns (DAMPs) whose production in the liver is induced during cellular stress, such as HMGB1, HSP70, HSP60, can trigger similar or complementary intracellular activation pathways, resulting in (necro-) inflammation or hepatocyte apoptosis in a steatotic liver leading to NASH^20,21^.

The role of specific macro- and likely micronutrients is becoming recognized as important modulators of NAFLD and NASH pathogenesis, either promoting or preventing disease^22,23^. We recently identified a common nutritional family of proteins, wheat amylase-trypsin inhibitors (ATI), as activators of toll like receptor 4 (TLR4) on monocytes, macrophages and dendritic cells^24,25^. ATI that represent 2–4% of the wheat protein are largely resistant to baking and intestinal proteolysis, and their ingestion promoted intestinal inflammation in a model of inflammatory bowel disease^25^, and worsened nutritional and inhalational allergies^26,27^. Notably, innate immune cell activation was higher in the mesenteric lymph nodes than in the gut lamina propria, suggesting a rapid propagation of the inflammatory signal to the periphery, likely by emigration of intestinal migratory macrophages-dendritic cells from the intestine shortly after their contact with ATI^25^.

We therefore explored the effect of nutritional ATI, as key proinflammatory micronutrients in the common staple wheat, on the severity of steatosis, metabolic derangement, and especially NASH and liver fibrosis in a dietary mouse model of NAFLD that reflects major facets of the human disease. Our results show that mere feeding of ATI in calorically irrelevant amounts and equivalent to average human daily wheat ingestion promotes weight gain, hepatic steatosis, inflammation and fibrosis, as well as insulin resistance, coupled with expansion of visceral adipose tissue mass and inflammation.

## Materials and Methods

### Experimental animals

#### Animal ethical approval

Animal experiments were approved by the State of Rhineland-Palatinate and performed in accordance with institutional and legal guidelines for animal protection under the ethical committee of the Government of Rhineland Palatinate under the reference number 2317707/G12-1-007.

Mice were kept in a climate-controlled environment with twelve hours of light/dark cycle and access to food and water *ad libitum*.

#### ATI purification and bioactivity determination

ATI were purified to >70% by quantitative extraction of wheat flour using 50 mM ammonium bicarbonate, pH 7.8, and fractional precipitation with ammonium sulfate as described^25^. Gluten was purchased from Sigma (Lot#SLBD0196V). TLR4 stimulating bioactivity of purified ATI and of ATI in gluten was determined by a THP1 macrophage bioassay using IL-8 secretion as readout and subsequently transformed into μg of bioactive ATI. The amount of ATI subspecies (mainly dimeric 0.19 and tetrameric CM3) was confirmed by mass spectrometry^25^.

#### Diets and feeding schemes

8-week-old male C57BL/6 mice were fed a basal carbohydrate and protein defined diet (22.1% of weight as zein, the non-inflammatory protein from corn whose components otherwise show similarities to those of wheat gluten), supplemented with amino acids, vitamins and minerals, combined with a low or high fat content (13 KJ% vs 53KJ% of calories as saturated fats) (prepared by Ssniff, Germany, Suppl. Table S1). Apart from the low fat diet (LFD) and high fat diet (HFD) alone, groups of mice received these diets with 30% of the zein being isocalorically replaced by crude wheat gluten (which contains 1.5% ATI) or with 0.7% of the zein being replaced by purified ATI (Supplementary Table S1). Thus, groups of 7–10 mice were kept on the following diets for 8 weeks: 1. LFD; 2. HFD; 3. HFD with gluten that contains ATI (HFD/G/ATI); 4. HFD with purified ATI and no gluten (HFD/ATI). During the feeding period, diet consumption was monitored thrice weekly. Mice were anesthetized by intraperitoneal injection of a mixture of 100 mg ketamine and 16 mg xylazine per kg body weight for sacrifice and blood collection via cardiac puncture. The liver, mesenteric, epididymal and inguinal adipose tissues were weighed and equal parts of liver and epididymal adipose tissue were fixed in OCT media (Medite, Burgdorf, Germany), in neutral-buffered formalin, or snap frozen in liquid nitrogen and kept at −80 °C.

### Intraperitoneal glucose tolerance test (IPGTT)

The IPGTT test was performed as described^28^. Briefly, immediately before sacrifice mice were transferred to clean cages without access to food but with drinking water ad libitum for 8–10 h. Then a 20% solution of 2 g glucose/kg of body weight was injected intraperitoneally. Blood samples were drawn from the tail vein immediately before and 15, 30, 60, and 120 min after the glucose challenge.

### Serum analyses

Serum alanine aminotransferase (ALT) and triglycerides were determined by the Central Clinical Laboratory of the University Medical Center Mainz with standardized and regularly validated clinical laboratory methods.

#### Fasting insulin and HOMA-IR index

Insulin serum levels were measured via enzyme-linked immunosorbent assay (ELISA) following the manufacturer’s instructions (DRG Instruments, Marburg, Germany). The homeostatic model assessment (HOMA)-IR index was determined as described elsewhere^29^, with some modifications. Briefly, after 8 weeks on the HFD and HFD/G/ATI, mice were fasted for 6–8 h, blood was drawn to measure glucose and insulin levels. The HOMA-IR index were calculated as described^30^, using the formula. HOMA IR = serum insulin (mmol/L)*(blood glucose (mmol/L)/22.5, as a surrogate for IR.

#### Hepatic collagen content determination

Hydroxyproline (Hyp), an amino acid that is unique to collagens and that represents roughly 10% of most collagen types, was quantified in  100–200 mg samples from 2 different liver lobes per animal after hydrolysis in 6 N HCl for 16 hours at 110 °C as described^31^ yielding relative Hyp (mg/g liver). Total collagen content was calculated based on individual liver weights and the corresponding relative HYP content^31^.

#### Evaluation of liver steatosis and inflammation

Liver sections (7 µm) were fixed in Tissue Tek (SAKURA, The Netherlands) that provide optimum cutting temperature (OCT) and stained with Sudan III (Sigma, Steinheim, Germany). Liver 7 µm cryosections were air dried at room temperature for 5 to 10 min and incubated in 0.3% Sudan III in 70% ethanol for 30 min, rinsed in distilled water and stained with hematoxylin for 3 min. After washing in water, the stained sections were mounted with glycerol resinous medium and viewed in a Zeiss Axio Imager AX10 microscope at magnification 40x. A series of random pictures covering most of the total tissue sections were generated, and Image J software was used to quantify liver fat. Paraffin-embedded liver sections (4 µm) were stained with haematoxylin and eosin (H&E, Sigma, Steinheim, Germany) and scored for liver inflammation and hepatocyte ballooning according to the NAS score^32^ adapted for mouse liver^33^.

#### Immunohistochemistry and quantitative morphometry

Formalin fixed, paraffin embedded tissue sections were dewaxed, rehydrated and rinsed in water; and antigens unmasked by boiling in 10 mM Na-citrate buffer (pH 6.0) for 30 min. To block endogenous peroxidase, sections were incubated in 3% H_2_O_2_ for 10 min, followed by rinsing in water and incubation with 5% normal donkey serum for 30–45 min. Sections were incubated with primary antibodies to CD68 (1:100, Biozol, clone: FA-11), Ym1 (1:500, Stemcell, clone: 01404), CD3 (1:100, Abcam, 28364) and α-SMA (1:500, Abcam, clone: E184 overnight at 4 °C, followed by biotinylated rabbit anti-mouse IgG (1:500, Vector Labs, BA-1000) for 30 minutes at room temperature, signal amplification with avidin-biotin complex, color development via diaminobenzidine (Vectastain ABC kit, Vector Laboratories, USA) and counterstaining with hematoxylin. For collagen staining, tissue sections were incubated with 0.1% Sirius red (SR) in saturated picric acid for 45 min, washed twice in 0.05% acetic acid and water followed by dehydration in ascending concentrations of isopropanol and finally immersion in xylene. SR stained sections were mounted with resinous medium, covered and visualized in a Zeiss Axio Imager AX10 Microscope with the appropriate filters. Representative images of 10 random high-power fields per liver were taken with an AxioCamMRc5 camera and quantitative morphometry was performed using the Image J software (National Institute of Health, Bethesda, Maryland, USA). Frozen intestinal 7 µm sections from the terminal ileum were formalin fixed for 5 to 10 minutes. Tissue was blocked with 5% normal donkey serum, and subsequently incubated with primary antibodies of to CD68 (1:100, Biozol, clone: FA-11), CD86 (1:100, Abcam, cat no: ab119857), and MHC-II (1:100, Abcam, Cat no: 180779) for 2 hrs at room temperature and finally incubated with respective Alexa-flour 488 labelled secondary antibodies. Stained liver and intestinal sections were visualized and a series of random pictures of perpendicularly oriented sections were taken using a Zeiss Axio Imager AX10 Microscope at magnification 40x and analyzed morphometrically with Image J software as for SR stained sections.

### Quantitative analysis of gene expression

Total RNA was extracted from livers, epididymal adipose tissue and from intestinal tissues with Ribozol (Amresco, Solon, USA), and complementary DNA was synthesized from 0.5 µg total RNA by reverse transcription using a cDNA SuperMix reverse transcription kit (Quanta, Gaithersburg, USA) with random hexamer primers. Quantitative real-time PCR (qPCR) was conducted in a Step OnePlus Real-Time PCR System using a commercially available SYBR Green qPCR system (Life technologies, Darmstadt, Germany) with specific primers for target genes as described in Supporting Table 2. Transcripts analysed were cd68, tnfa, il1a, il6, arg1, ym1, col1a1, tgfb1, timp1, mmp9, mmp12, and mmp13 (Supplementary Table 2). Relative expression of each gene was quantified by normalization to glycerinaldehyde-3-phosphat-dehydrogenase (GAPDH) mRNA expression.

#### Immune cell subset analysis via flow cytometry

Liver tissue was homogenized with the gentleMACS dissociator (MACS Miltenyi Biotec, Bergisch Gladbach Germany) and incubated with 0.4% collagenase IV (Sigma), 1.6 nM DNaseI (Applichem, Munich, Germany) in 154 mM NaCl, 5.6 mM KCl, 5.5 mM glucose, 20.1 mM HEPES, 25 mM NaHCO_3_, 2 mM CaCl_2_, 2 mM MgCl_2_, pH7.4, for 30 min at 37 °C. Tissue homogenates were filtered through a 100 μm cell strainer (BD Bioscience, Leipzig, Germany and centrifuged at 21 × g for 4 min to remove contaminating hepatocytes. The supernatant was centrifuged at 300 × g for 10 min and the pellet resuspended in red blood cells lysis solution (Ebioscience) for 10 min at RT and centrifuged for 10 min at 300 g. Non-specific antibody binding sites were blocked with anti-Fc receptor IgG (1:100, BD Bioscience, clone: 2.4G2) for 10 min, followed by centrifugation and subsequent incubation of the pellet with target FACS antibodies to CD45 (1:100, clone:30-F11), CD11b (1:100, clone: M1/70), Ly6C (1:100, clone: HK1.4) and F4/80 (1:100, clone: BM8). After antibody staining, cells were fixed with 1% formaldehyde buffer. Data were acquired on a FACS Canto II (BD Bioscience) and analyzed using the FlowJo 7.6 software (TreeSta). The gating strategy is shown in Supplementary Fig. 1.

### Statistical analysis

Data were analysed using Graphpad Prism 5.0 (GraphPad software, La Jolla, USA). Binary comparisons were done with the unpaired t test, and ANOVA was employed for multiple group comparisons. The data were expressed as the mean and standard error of the mean. Differences between groups with p < 0.05 were considered significant.

## Results

### ATI in a gluten matrix or alone promote insulin resistance

**Figure 1:**
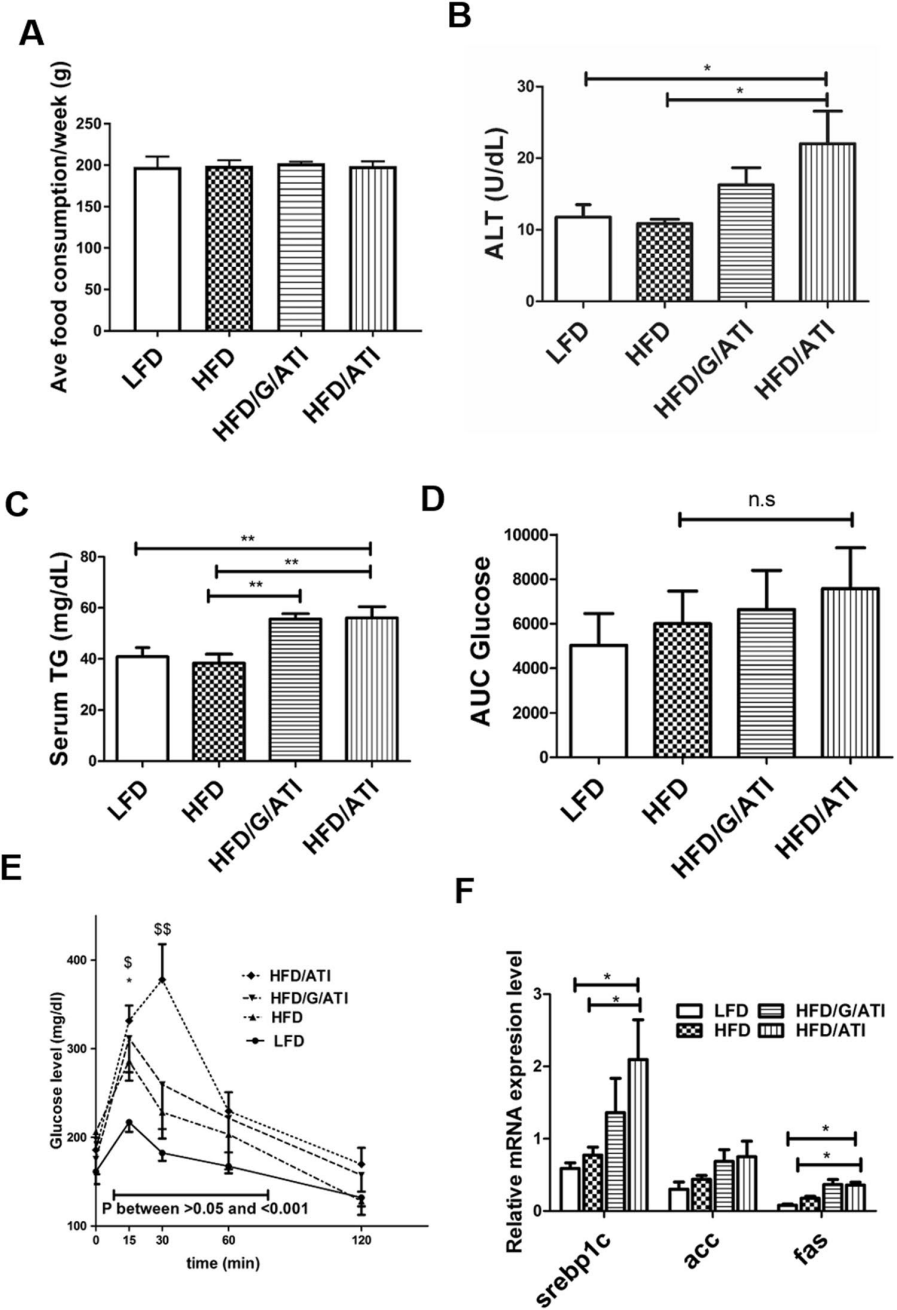
Enhanced insulin resistance, higher serum transaminases and increased lipogenic gene expression in mice fed ATI. Age-matched, wildtype mice were fed four different diets for 8 weeks. A low fat diet (LFD), a high fat diet (HFD), a HFD/G/ATI diet (G, 30% of protein as gluten, and 0.45% as ATI) and a HFD/ATI diet (0.7% of protein as ATI). (**A**) Average food consumption per week (**B**) Serum ALT (**C**) Serum triglycerides. (**D**) Average AUC (area under the curve) of the aforementioned groups (**E**) Intraperitoneal glucose tolerance test (IPGTT), in the same dietary groups as shown in A. (**F**) Hepatic transcript levels of srebp1c, acc, fas. Comparisons by ANOVA; data are means ± SEM for 7–10 mice per group; *p < 0.05, **p < 0.01; ^$^p < 0.05, ^$$^p < 0.01.

Mice were subjected to a short-term high fat diet (HFD, 8 weeks) with defined carbohydrates and zein from corn supplemented with essential amino acids, as defined protein and nutrient source. Subgroups received a low-fat diet (LFD), or the HFD, either with 30% of the zein being isocalorically replaced by gluten (which contains 1.5% as ATI, resulting in 0.45% of total protein as ATI, HFD/G/ATI), or the HFD diet mixed with purified ATI (0.7% of total protein as ATI, HFD/ATI), as detailed in Supplementary Table 1. These amounts of ATI compare well with the average human daily consumption, which is around 150 g of processed wheat flower per day in the Western or middle Eastern diet^25^. Mice fed the HFD with ATI exhibited significantly higher ALT and triglyceride levels than their HFD controls. Total food consumption was comparable among the groups, while relative food consumption in the HFD/ATI-fed mice was even reduced compared to the HFD controls, due to their increased body weight (Fig. 1A–C, Supplementary Fig. 1A). Moreover, with an intraperitoneal glucose tolerance test the HFD/ATI > HFD/G/ATI fed mice showed significantly elevated early blood glucose levels, and thus incipient insulin resistance (IR) compared to the mice on the HFD alone (Fig. 1D), with a nearly significantly increased area under curve up to 120 min (Fig. 1E). We also determined the HOMA-IR index in a separate experiment. Here, fasting insulin levels and glucose were nearly significantly elevated, while the HOMA-IR index was significantly increased in the HFD/G/ATI vs the HFD group, confirming that the inflammatory trigger of nutritional ATI promotes IR in this NASH model (Supplementary Fig. 1B,C).

Notably, in these studies, and in the following experiments, the strongest effect was observed in mice that consumed the higher amount of purified ATI (0.7% of total protein) compared to mice that consumed 0.45% ATI combined with gluten, despite the massive surplus of gluten in the latter group (30% of total protein). This confirms our prior data that it is the TLR4 activating ATI and not the prevalent gluten proteins in wheat that stimulate innate immune cells and thus promote inflammation^24,25^. There was a good correlation between the weight of mesenteric and epididymal adipose tissue, both considered unfavourable fat depots, in all groups except for a trend only in the HFD/G/ATI fed group (Supplementary Fig. 3).

### Nutritional ATI promote hepatic steatosis and inflammation

**Figure 2:**
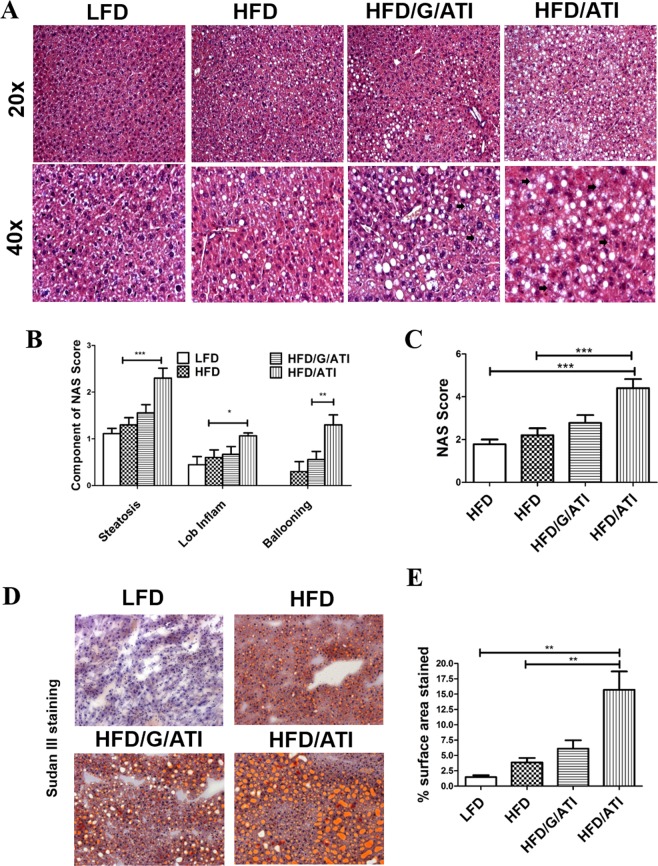
ATI feeding promotes hepatic steatosis and inflammation. (**A**) Representative images of H&E stained liver sections (original magnifiction 20x and 40x). (**B,C**) Grading of steatosis, lobular inflammation and hepatocyte ballooning (arrows), according to the NAFLD Activity Score (NAS). (**D**) Frozen liver sections stained with Sudan III (original magnification 20x). **(E)** Quantification of % of Sudan III stained area. Comparisons by ANOVA; data are means ± SEM for 5 representative sections per mouse and 7–10 mice per group; *p < 0.05, **p < 0.01, ***p < 0.001.

All components of the histological non-alcoholic steatohepatitis (NAS) score^32^, as adapted for mouse liver^33^, including steatosis, inflammation and hepatocyte ballooning, scored significantly higher in the HFD plus ATI vs the HFD mice, again with highest NAS values in the mice fed 0.7% of pure ATI vs. 0.45% of ATI in a gluten matrix (Fig. 2A–C). The semiquantitative steatosis data were confirmed using lipid morphometry on Sudan red stained liver sections; with an almost three-fold increase in the HFD/ATI vs the HFD group (Fig. 2D,E). Furthermore, there was a significant increase in the expression of genes central for lipogenesis in the ATI/HFD vs the HFD group, with a trend in the HFD/G/ATI group, i.e., mice that consumed a lower amount of ATI (Fig. 1F).

### Nutritional ATI stimulate hepatic proinflammatory macrophage infiltration

**Figure 3:**
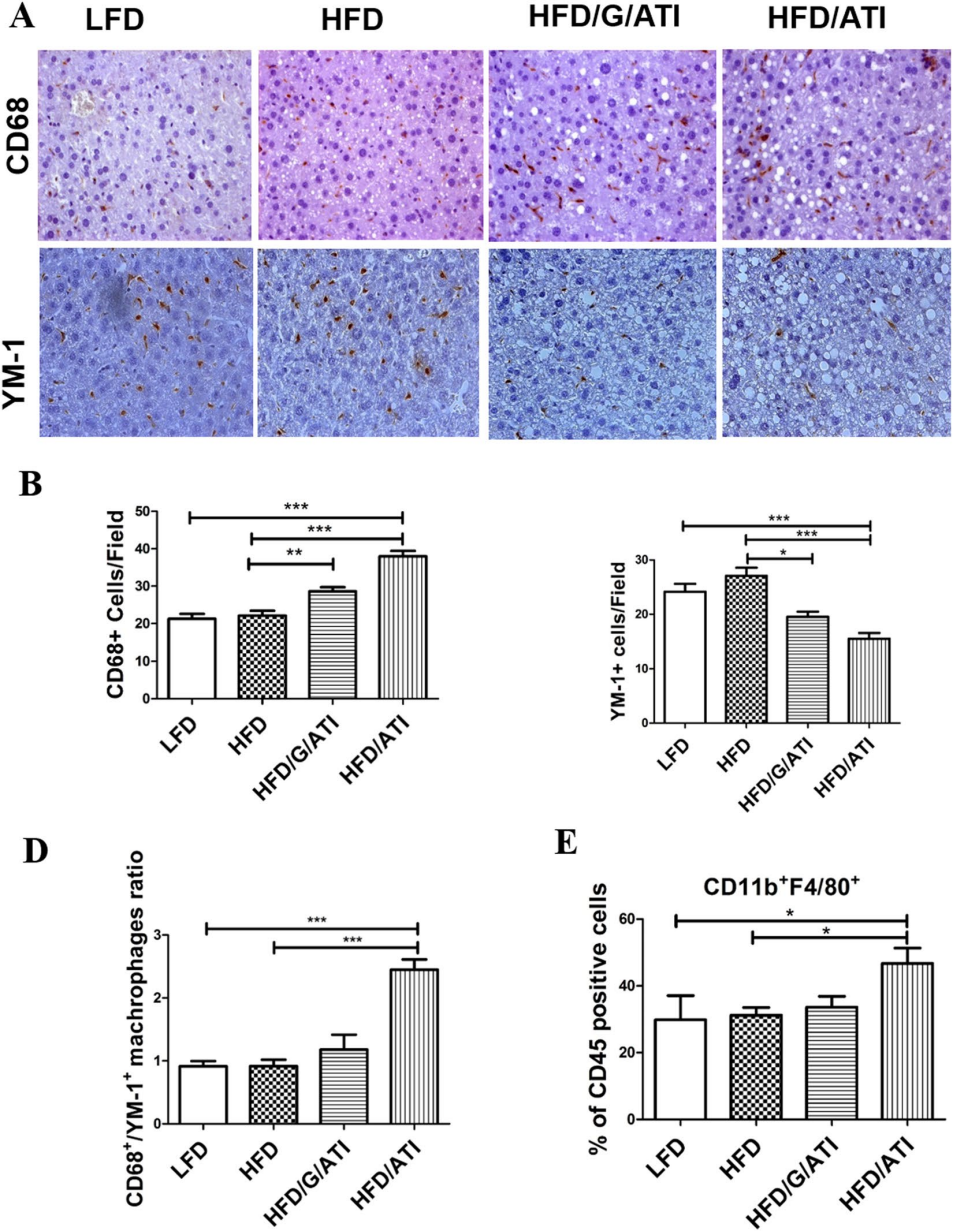
ATI feeding increases liver macrophage numbers and their M1- vs M2-type polarization. (**A**–**C**) Immunohistochemistry and quantitative morphometry for CD68 and YM-1 positive cells (original magnification 40x). (**D**) Ratio of total (CD68+) vs M2-type (Ym-1+) macrophages. (**E**) CD11b+ F4/80+ macrophage subset (% of CD45 positive total immune cells) as determined by FACS analysis. Comparisons by ANOVA; data are means ± SEM for 10 representative sections per mouse and 7–10 mice per group; *p < 0.05, **p < 0.01, ***p < 0.001.

Quantitative immunohistochemistry revealed that cells expressing CD68 (Fig. 3A,B), a general macrophage marker, were increased, whereas cells expressing YM-1 (Fig. 3C), a putative M2 macrophage marker, were decreased in livers of HFD/ATI and HFD/G/ATI fed mice compared to mice on the HFD alone, suggesting an increase of proinflammatory M1 over supposedly anti-inflammatory M2-type (YM-1 positive) macrophages (Fig. 3D). This result was supported by FACS analysis (gating strategy see Supplementary Fig. 2) of liver immune cells which showed a significantly increased population of CD11b+ F4/80+ liver resident macrophages in the HFD/ATI fed mice (Fig. 3E). Moreover, there was a significant increase in CD3+ T-cells (Supplementary Fig. 4) in ATI plus HFD vs HFD group.

**Figure 4:**
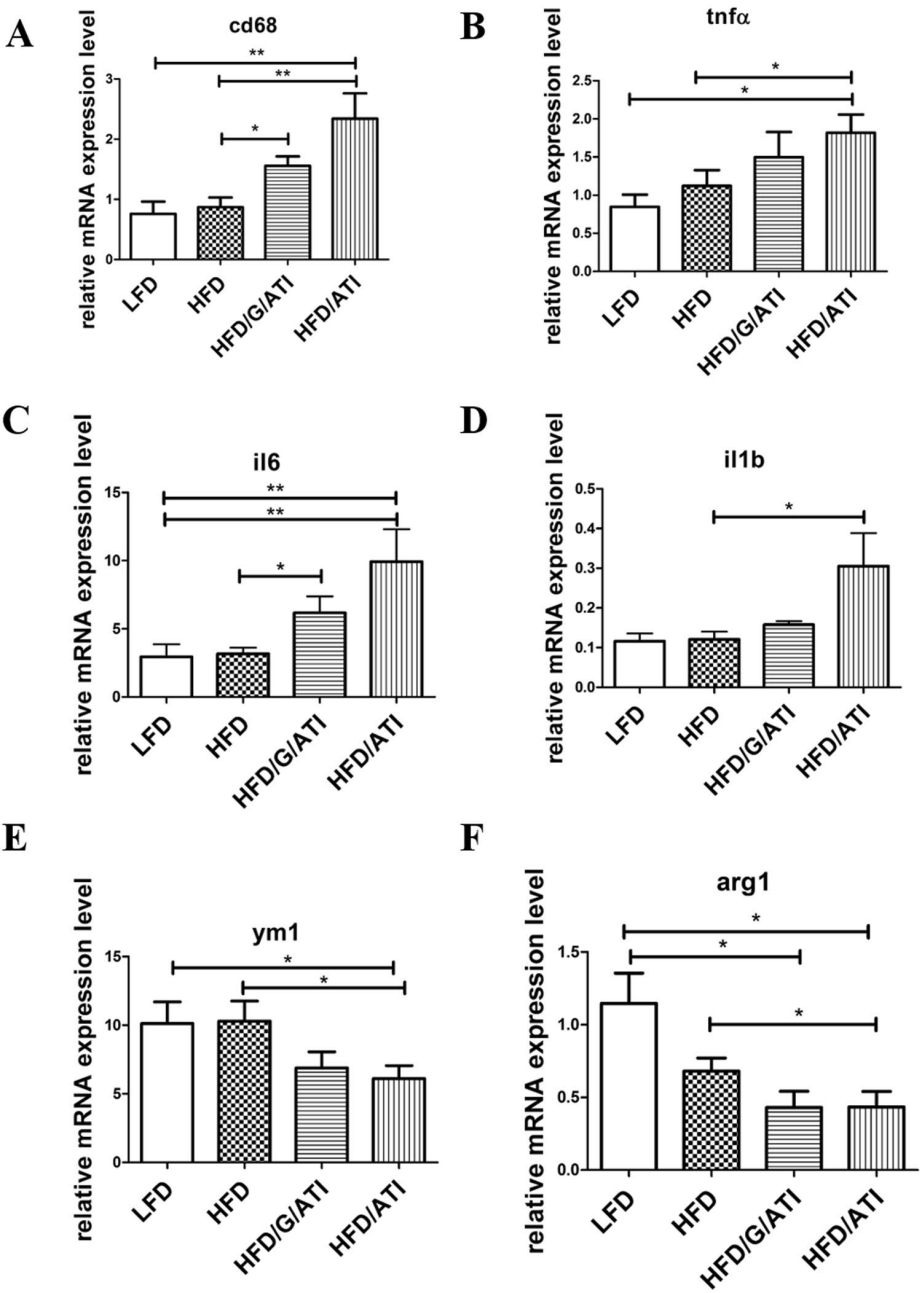
ATI feeding increases hepatic pro-inflammatory and macrophage M1- vs M2-type gene expression. (**A**–**F**) Hepatic transcript levels of cd68, tnfa, il1b, il6, arg1 and ym1. Comparisons by ANOVA; data are expressed as means ± SEM for 7–10 mice per group; *p < 0.05, **p < 0.01.

In line with increased inflammatory macrophage numbers, transcript levels of cd68 (total macrophages) and pro-inflammatory cytokines (tnfa, il1b, il6) were upregulated, while transcript levels of M2-type macrophage markers (arg1, ym1, cd206) were downregulated in livers of the HFD/ATI vs HFD fed mice (Fig. 4A–F and Supplementary Fig. 5A).

### Promotion of hepatic fibrogenesis by nutritional ATI

**Figure 5:**
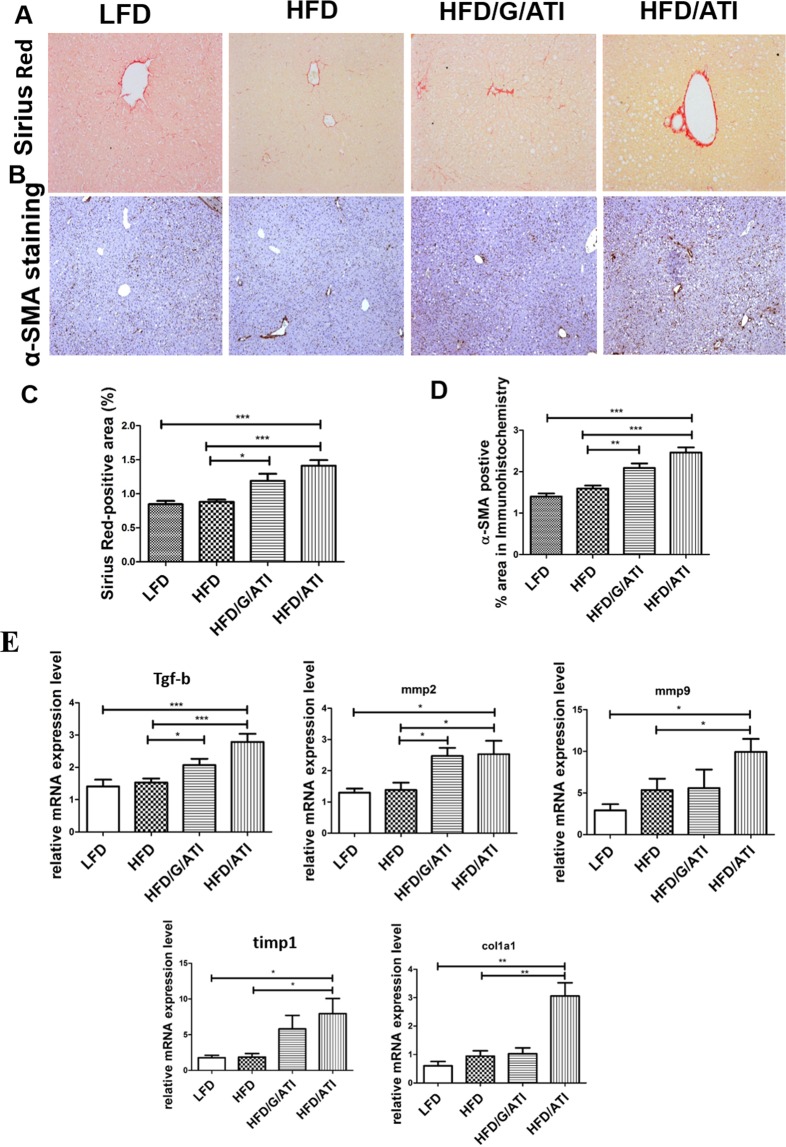
ATI feeding promotes hepatic fibrogenesis. (**A**) Sirius Red and (**B**) α–SMA immunohistochemistry and (**C,D**) quantitative morphometry (original magnification 20x). Hepatic transcript levels of tgfbeta, mmp2, mmp9, mmp13, col1a1, timp1. Comparisons by ANOVA; data are expressed as means ± SEM of 5 representative sections per mouse and 7–10 mice per group; *p < 0.05, **p < 0.01, ***p < 0.001.

The employed HFD model induces only very mild liver fibrosis, as reflected by only a trend of increased total hydroxyproline (HYP), a quantitative measure of biochemical collagen, in all experimental groups (Supplementary Fig. 5B). However, morphometry of Sirius red stained collagen, a method that better captures functionally relevant parenchymal vs the denser; but less relevant portal collagen deposition, was significantly increased by more than 50% in the HFD/ATI-fed mice compared to mice that received the HFD alone (Fig. 5A,C). This was paralleled by an increase of α-SMA positive cells representing a subset of activated hepatic stellate cells and myofibroblasts (Fig. 5B,D). Moreover, dietary ATI significantly upregulated hepatic gene expression of fibrosis related transcripts, such as tgfb1, mmp2 and mmp9, timp1, col1a1 (Fig. 5E) and mmp13 (Supplementary Fig. 5C). Collectively, amounts of ATI in the diet that resemble average daily consumption in man significantly promoted hepatic inflammation and fibrogenesis, likely via enhanced recruitment and activation of proinflammatory vs putatively anti-inflammatory monocytes-macrophages.

### Dietary ATI fuel adipose tissue inflammation

**Figure 6:**
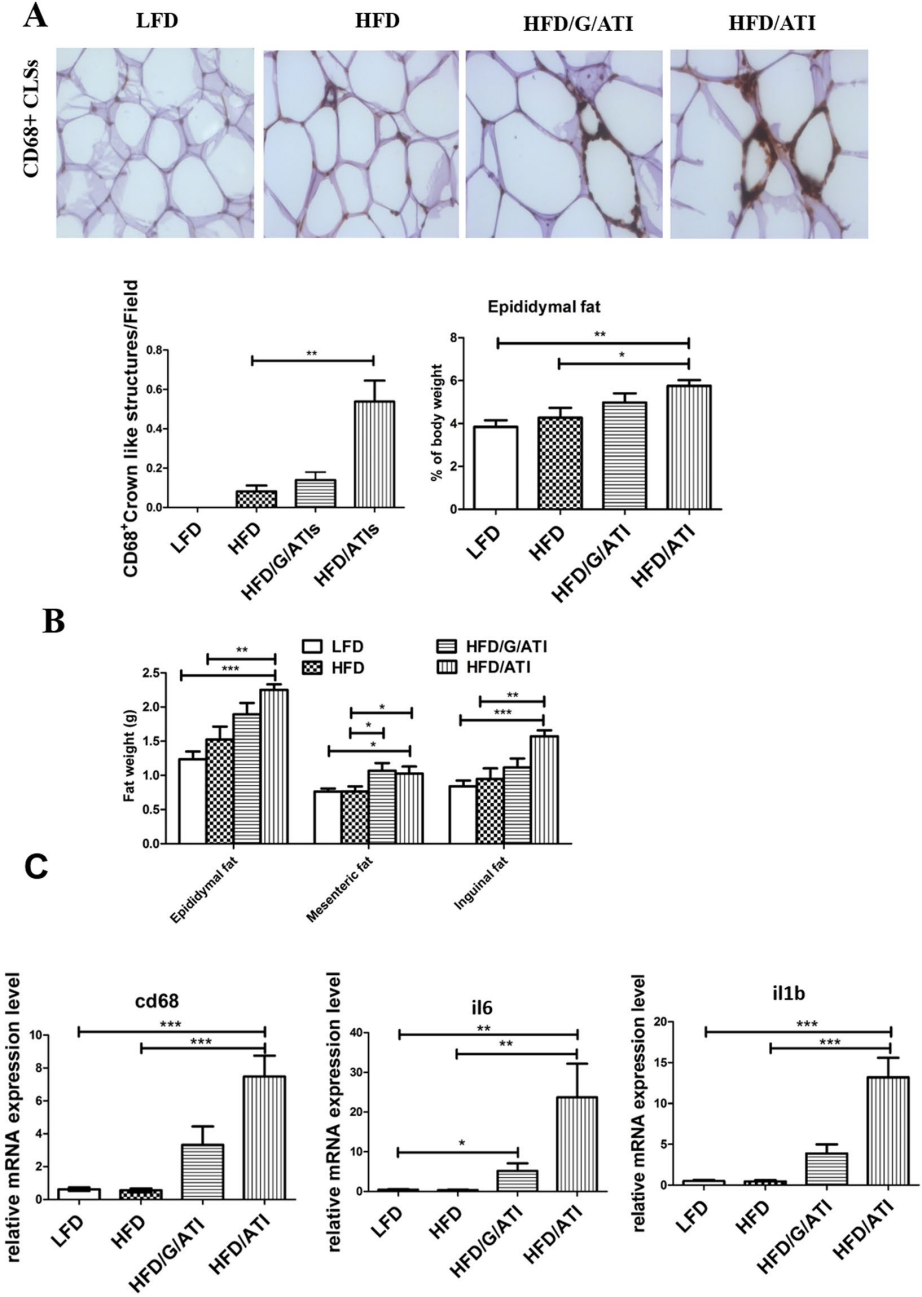
Nutritional ATI promote central adipose tissue inflammation. (**A**) Crown like structures (CLS = accumulation of macrophages) in CD68+ stained sections of epididymal adipose tissue in the 4 experimental groups (original magnification 40x), the number of CD68+ CLS as determined by morphometry, and epididymal fat as % of body weight. (**B**) fat weights, and (**C**) transcript levels of cd68, il6 and il1b. Comparisons by ANOVA; data are means ± SEM for 10 representative sections per mouse and 7–10 mice per group; *p < 0.05, **p < 0.01, ***p < 0.001.

Dietary ATI dose-dependently, i.e. HFD/ATI > HFD/G/ATI (0.45% vs. 0.7% of protein as ATI), worsened inflammation of epididymal adipose tissue which is considered the murine equivalent of visceral adipose tissue in man^34,35^, as revealed by a striking increase of CD68+ crown like structures (Fig. 6A) that represent accumulations of inflammatory macrophages^36^. There was a significant increase of inguinal, mesenteric, and epidydimal fat, the latter two fat depots representing central adipose tissue^34–36^ (Fig. 6B). Moreover, a significant upregulation of cd68, il1b and il6 transcripts in ATI/HFD vs the HFD alone fed mice were observed (Fig. 6C).

### ATI feeding induces intestinal inflammatory markers and dendritic cell activation and maturation

**Figure 7:**
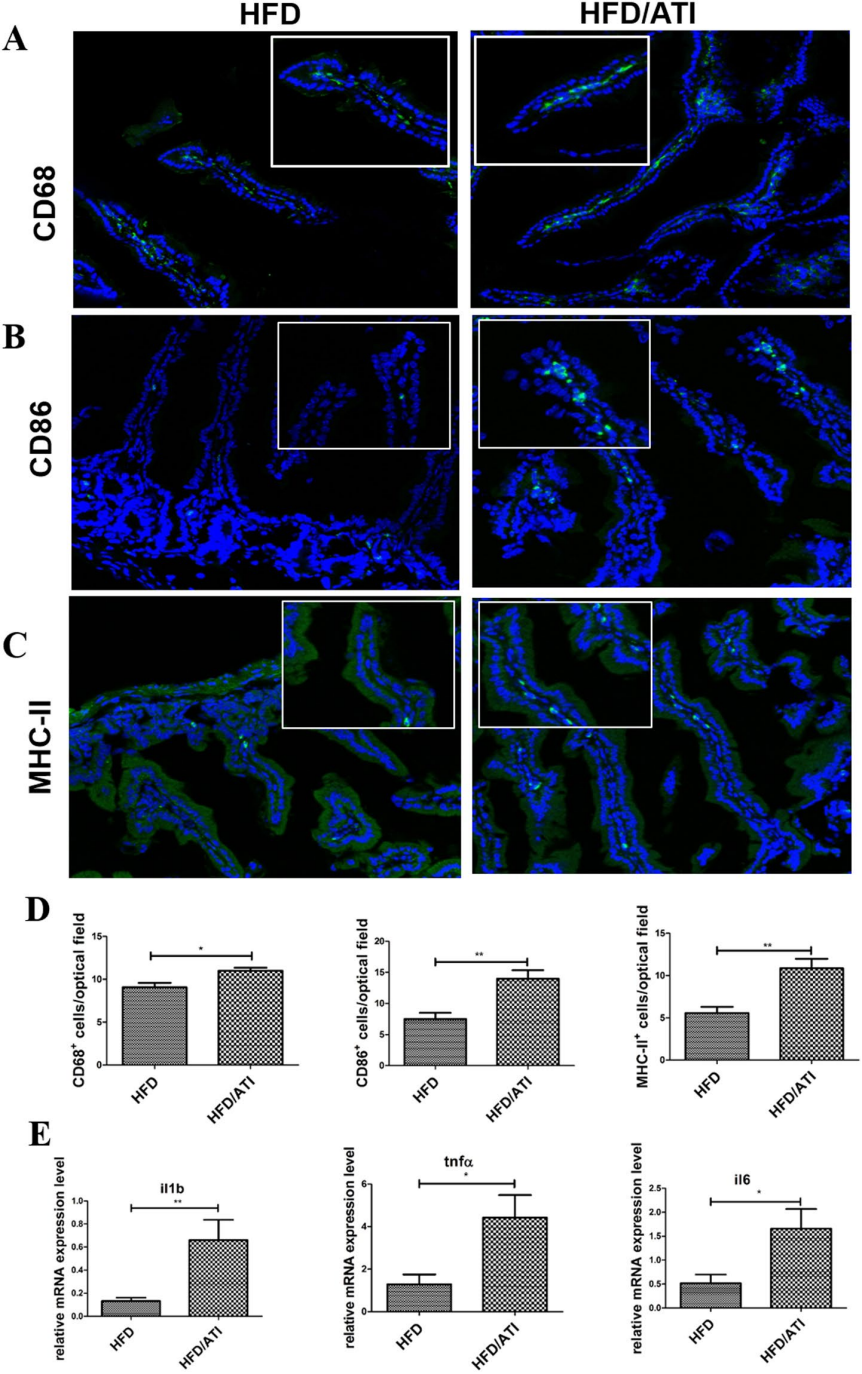
ATI feeding increases intestinal macrophage and dendritic cell activation and maturation. (**A–C**) CD68, CD86 and MCH-II expressing cells in the terminal ileum; scale bar: 100 and 50 µm. (**D**) Morphometric quantification of CD68, CD86 and MHC-II positive cells. (**E**) Transcript levels of il1b, tnfα and il6. Comparisons by ANOVA; data are expressed as means ± SEM of 6 mice per group and 5 representative sections per mouse; *p < 0.05, **p < 0.01, ***p < 0.001.

Distal small intestinal tissue  of mice fed the HFD plus ATI harboured significantly increased numbers of CD68+, CD86+, and MHC-II+ myeloid cells (Fig. 7A–D) compared to mice fed the HFD alone. Moreover, intestinal il1b, tnfa, and il6 transcript levels were significantly increased in the HFD/ATI vs the HFD group (Fig. 7E).

## Discussion

The liver (in addition to the intestine) acts as the first line of defense against gut-derived antigens^17^. We previously demonstrated that nutritional wheat ATI trigger an innate immune response in intestinal myeloid cells via the TLR4-MD2-CD14 complex, thus exacerbating colitis^25^. Moreover, inhalational as well as nutritional allergic T cell responses are promoted by ingestion of ATI^26,27^. In general, our results indicate that nutritional ATI serve as adjuvant for intestinal and extra-intestinal inflammatory diseases, with propagation of the intestinal inflammatory signal to the mesenteric lymph nodes and intestinal and peripheral target organs, possibly by migration of the ATI-activated myeloid cells^24,37,38^. Here, we provide evidence that nutritional ATI increase visceral adipose tissue inflammation and the NAFLD phenotype in mice fed a high fat diet. We also confirm our prior results that indicated that ATI, a minor protein constituent of wheat and related cereals, and not the abundant gluten proteins harbor the innate immune activating activity of wheat, in contrast to celiac disease, where patients with a necessary but not sufficient genetic predisposition (presennce of HLA-DQ2 or -DQ8) develop a gluten-specific intestinal T cell response that leads to severe intestinal inflammation with villous atrophy and crypt hyperplasia^24,39^. Different from celiac disease, ATI elicit a modest immune response in the gut, with a mild increase in CD68+ macrophages and a modest increase of lamina propria dendritic cells/macrophages expressing CD86 and MHC class II, as also confirmed in the present report. However, upon intestinal ATI exposure, a strong adjuvant immune response on general and antigen specific T cell activation was found *in vitro* and in mesenteric lymph nodes *ex vivo*^25–27^ indicating that ATI responses are largely extraintestinal, and explaining the more severe symptoms of so-called non celiac wheat sensitivity^37,40^.

Various etiological factors such as inflammation, aborted lipid metabolism and certain intestinal microbiota can promote insulin resistance (IR)^41^. IR at the level of adipose tissue and liver as considered an important cofactor for the development and progression of NAFLD^11^. Moreover, NAFLD and NASH are affected by genetic and especially environmental factors, especially nutrition. The nutritional effect is not only due to the mere caloric value of macronutrients, but likely includes certain micronutrients with immune-modulatory properties^10,42^. Here, we focus on ATI, a micronutrient with no relevant caloric value, that acts as cofactor for the development of IR, and especially the progression of NAFLD to NASH, a finding of high relevance in our increasingly wheat consuming societies. Thus, mice on a HFD supplemented with ATI in doses that are equivalent to those contained in average wheat-based diets significantly and dose-dependently developed IR, and importantly adipose tissue inflammation, as is functionally associated with NASH. Thus, ATI feeding caused an increase in ALT and triglyceride levels. In this line, all visceral adipose tissue compartments (epididymal, mesenteric, and inguinal) were significantly expanded in the ATI supplemented HFD fed mice compared to the HFD controls, with correlation between these visceral compartments. Notably, adipose tissue inflammation was dominated by macrophages, as exemplified by formation of crown-like structures (CLS) and pronounced expression of macrophage specific inflammatory genes^31–35,43^. We further showed that the increase of CLS goes along with a significant upregulation of genes reflecting pro-inflammatory M1-type macrophage activation (il1b, il6), in accord with prior data on adipose tissue inflammation in mice^44^. Thus, the significant upregulation of M1-type macrophages with pronounced formation of CLS demonstrates that ingestion of ATI promoted metabolic inflammation in the visceral adipose tissues.

As in man, the severity of adipose tissue inflammation correlated with the severity of NAFLD/NASH^45^ in our dietary mouse model. Upon histological assessment using the NAS score and its individual components (steatosis, lobular inflammation, ballooning) adapted to the rodent system^33,46^, liver injury was promoted, again dose-dependently, by ATI in mice fed the HFD. Here, increasing severity was not only documented by inflammatory infiltrates, but also by hepatocyte ballooning which is considered a hallmark of NASH, caused by cellular lipo-apoptosis which is a central driver of fibrosis progression^47^. Similar to visceral adipose tissue, hepatic inflammation in the ATI-fed mice was dominated by macrophages with a prominent M1-type (pro-inflammatory) over M2-type (putatively anti-inflammatory) phenotype. This could be illustrated by elevated numbers of CD68+ total and CD11b^+^F4/80^+^ resident liver macrophages, and a relative decrease of Ym-1+ M2-type macrophages in the livers of the ATI-HFD fed mice. Moreover, compared to mice fed the HFD without ATI, hepatic transcript levels of cd68 and the M1-type macrophage markers il6 and tnfa were highly upregulated, whereas the expression of putative anti-inflammatory M2-type macrophage markers (arg1, cd206, ym1) and of YM-1 protein were downregulated.

Importantly, although overall fibrosis was mild, mice on the HFD plus ATI developed clearly more histological fibrosis, as demonstrated by Sirius red morphometry and biochemical collagen quantification, with a higher expression of fibrogenesis-related genes, and an increased number of activated hepatic stellate cells/myofibroblasts, This is remarkable, since HFD feeding with or without ATI lasted only for 8 weeks, a time period which does not produce any fibrosis and only insignificant inflammation when no other damaging factors are present such as dietary choline deficiency or excess cholesterol^47–49^.

This can be explained by our prior data and also in the present report that show an increase in intestinal cells/macrophages expressing CD86 and MHC class II due to dietary ATI. It is likely that ATI activate intestinal monocytes-macrophages and dendritic cells to leave the GI tract and co-stimulate ongoing innate and T cell mediated immune pathology in the periphery. Here, the liver would be the first major organ to receive these pro-inflammatory intestinal signals. ATI further enhance these signals via cytokines and chemokines produced by the activated myeloid cells in the gut and/or after their migration to mesenteric or further peripherally located lymph nodes. This is supported by an observed 2–3 fold increase of circulating proinflammatory cytokines and chemokines like IL-6 and MCP-1 after a single oral gavage of ATI to normal mice or to mice with experimental inflammatory bowel disease^25^ Alternatively, the myeloid cells, after sensing the ATI, may migrate further towards the end-organ, here the liver (and adipose tissue), where they would directly promote metabolic inflammation, keeping in view the postulated nexus of inflammation mediated obesity and insulin resistance.

In summary, our study implicates a defined micronutrient, ATI, from the common staple wheat, to act as pro-inflammatory nutritional driver of adipose tissue inflammation, insulin resistance and NAFLD/NASH. This effect occurs at a daily intake that is comparable to average human consumption of wheat products. A clinical trial of the effect of wheat/ATI consumption on human NAFLD/NASH severity is planned.

## Supplementary information

Supplementary information
