# Supplementary material for: Dietary wheat amylase trypsin inhibitors promote features of murine non-alcoholic fatty liver disease
Source: Sci Rep. 2019 Nov 25;9:17463. doi: 10.1038/s41598-019-53323-x (PMC6877510; doi:10.1038/s41598-019-53323-x)
Supplement: Supplementary file 1 — Supplementary information [file 41598_2019_53323_MOESM1_ESM.pdf]

Dietary wheat amylase trypsin inhibitors promote features of murine non-alcoholic fatty liver disease

Muhammad Ashfaq-Khan[1], Misbah Aslam[1], Muhammad Asif Qureshi[1], Marcel Sascha Senkowski[1], Shih Yen-Weng[1], Susanne Strand[2], Yong Ook Kim[1], Geethanjali Pickert[1], Jörn M. Schattenberg[2], Detlef Schuppan[¥1,]

[1]Institute of Translational Immunology and Research Center for Immune Therapy, University Medical Center, Johannes Gutenberg University Mainz;

[2] Department of Medicine I, University Medical Center, Johannes Gutenberg University, Mainz, Germany

Address for correspondence:

Detlef Schuppan, MD, PhD,

Institute of Translational Immunology

University Medical Center

Langenbeckstr. 1

55131 Mainz

Phone (fax): +49-6131-17-7356 (57).

Email: detlef.schuppan@unimedizin-mainz.de

LEGENDS TO SUPPLEMENTARY TABLES AND FIGURES

Supplementary Fig.1. Food intake and HOMA-IR.

A: Food intake normalized to body weight, B, fasting insulin levels C, HOMA-IR. Comparisons in A by ANOVA; data are expressed as means ± SEM for 7-10 mice per group; *p<0.05, **p<0.01, ***p<0.001. Binary comparisons in B and C were done with the unpaired t-test. Data are expressed as means ± SEM for 7-10 mice per group; *p<0.05, **p<0.01, ***p<0.001.

Supplementary Fig.2. Gating strategy for FACS.

Analysis was done after exclusion of cell debris and removal of aggregated cells. Then the selection of live cells was followed by sorting for $CD45^+$ immune cells. From the CD45+ immune cells, $Ly6C^+$ and $CD11b^+$ double positive cells were selected, followed by further gating for $LY6C^+$ $CD11b^+$ $F4/80^+$ cells which represents the target cell population of interest.

Supplementary Fig.3. Correlation among different fat depots in all groups.

Correlation between mesenteric and epididymal fat mass between groups. Comparisons by ANOVA; data are expressed as means ± SEM for 7-10 mice per group; *p<0.05, **p<0.01, ***p<0.001.

Supplementary Fig.4. ATI feeding increases CD3+ T cells in HFD fed mice.

Comparisons by ANOVA; data are expressed as means ± SEM for 6 mice per group and 5 representative sections per mouse; *p<0.05, **p<0.01, ***p<0.001.

Supplementary Fig.5. Hepatic hydroxyproline content and gene expression analysis. A,C: Quantitative PCR, B: Quantification of total liver collagen (hydroxyproline). Comparisons by ANOVA; data are expressed as means ± SEM for 7-10 mice per group; *p<0.05, **p<0.01, ***p<0.001.

Supplementary Table 1. Diet composition.

Supplementary Table 2. List of PCR primers.

Suppl. Fig 1: Normalized food intake, Fasting insulin level and HOMA-IR index

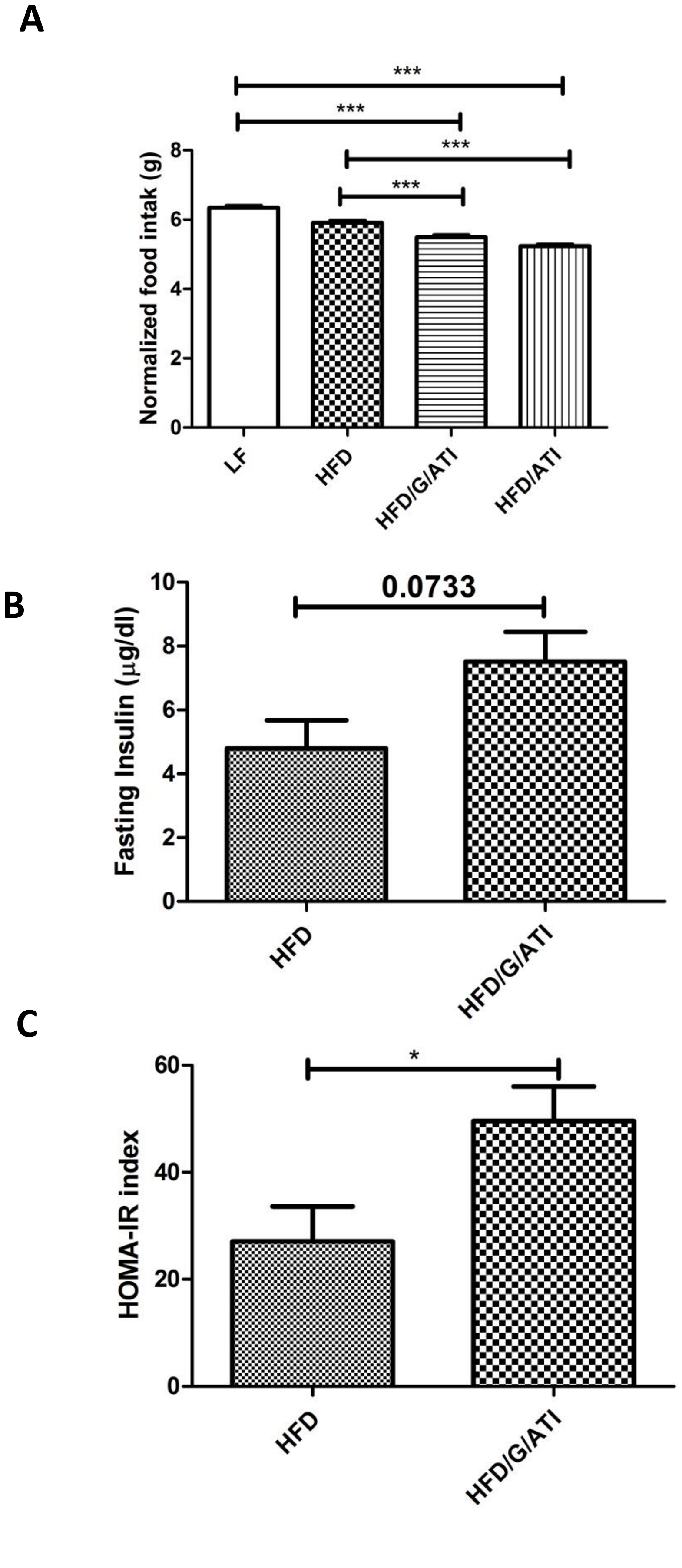

Suppl. Fig 2: Gating Strategy

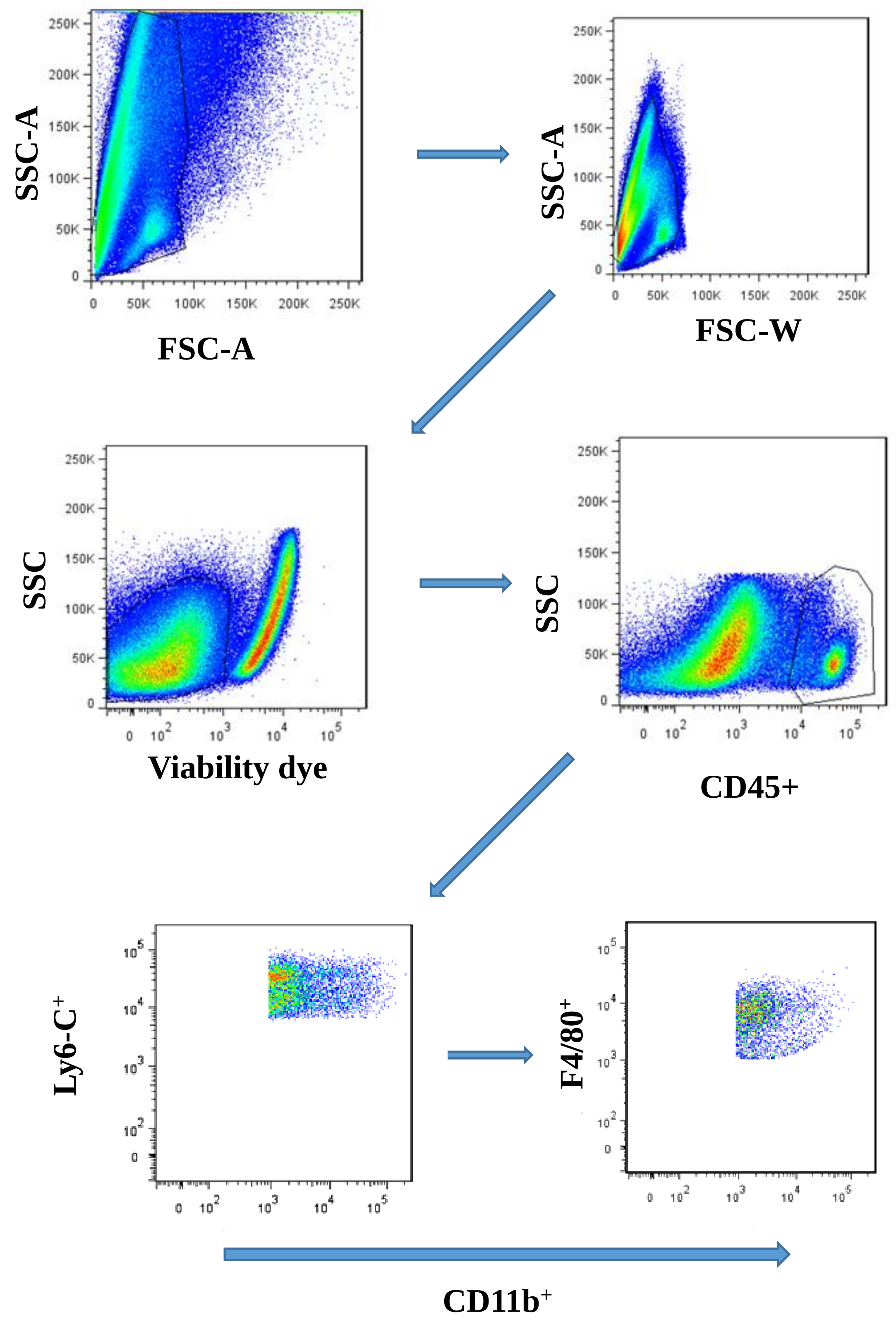

Suppl. Fig 3

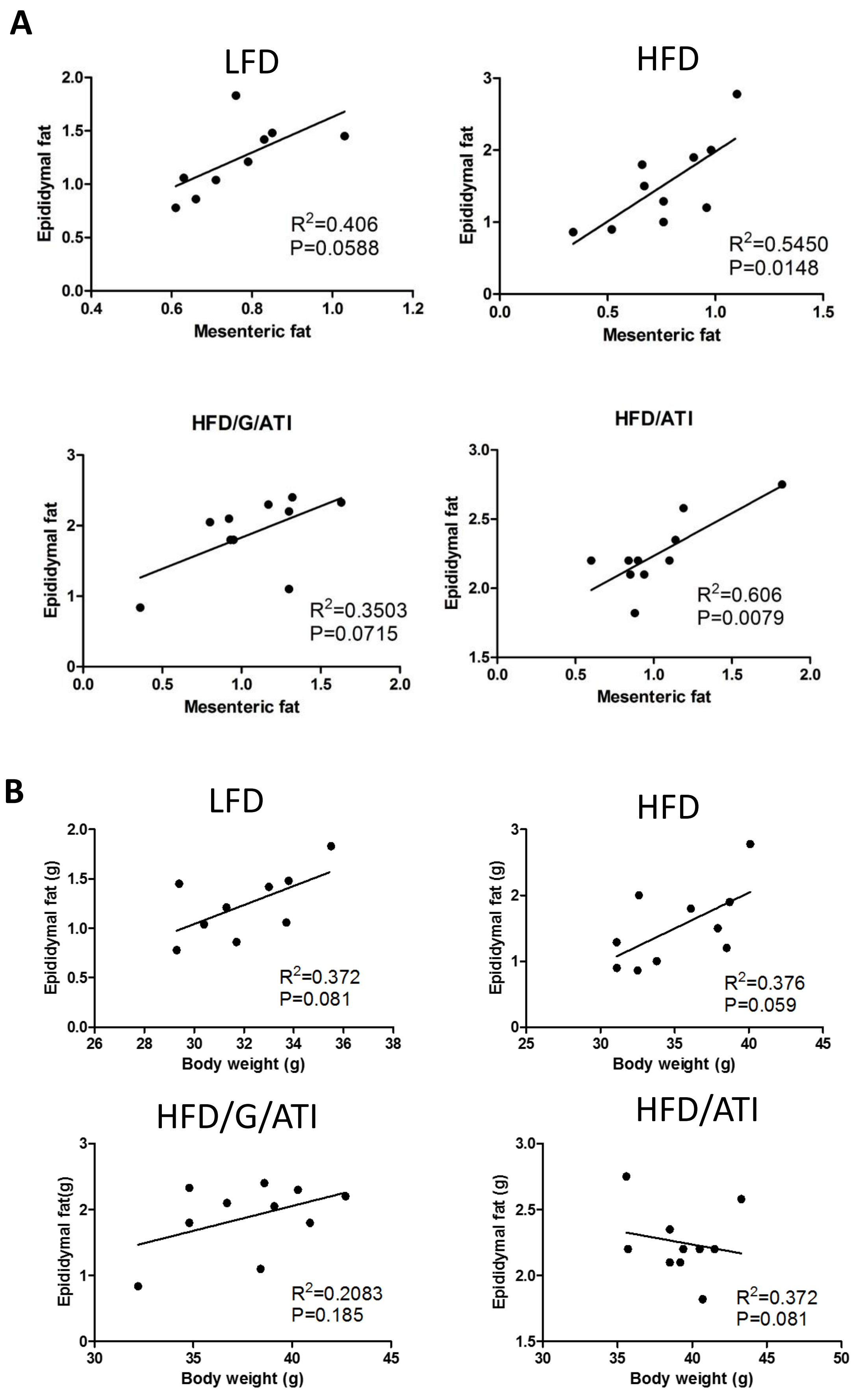

A
LFD
Epididymal fat
Mesenteric fat
R2=0.406
P=0.0588
HFD
R2=0.5450
P=0.0148
HFD/G/ATI
R2=0.3503
P=0.0715
HFD/ATI
R2=0.606
P=0.0079
B
LFD
Epididymal fat (g)
Body weight (g)
R2=0.372
P=0.081
HFD
R2=0.376
P=0.059
HFD/G/ATI
Epididymal fat(g)
R2=0.2083
P=0.185
HFD/ATI
R2=0.372
P=0.081

Suppl. Fig 4

A

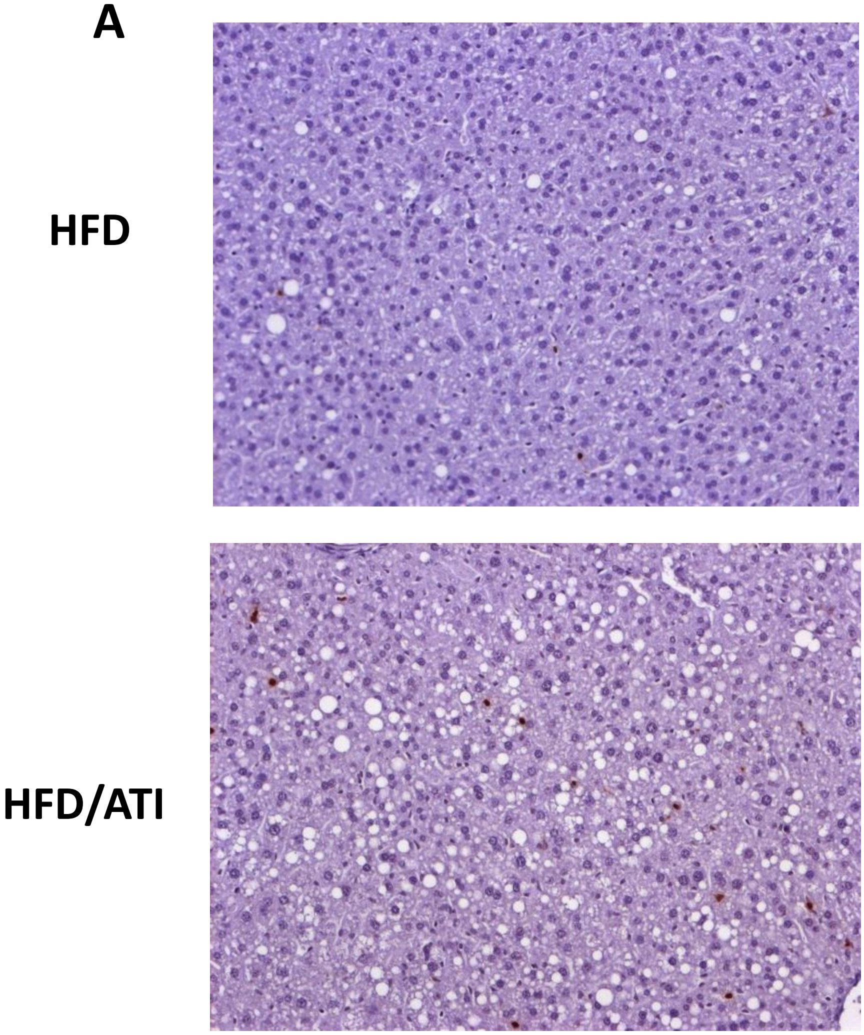

B

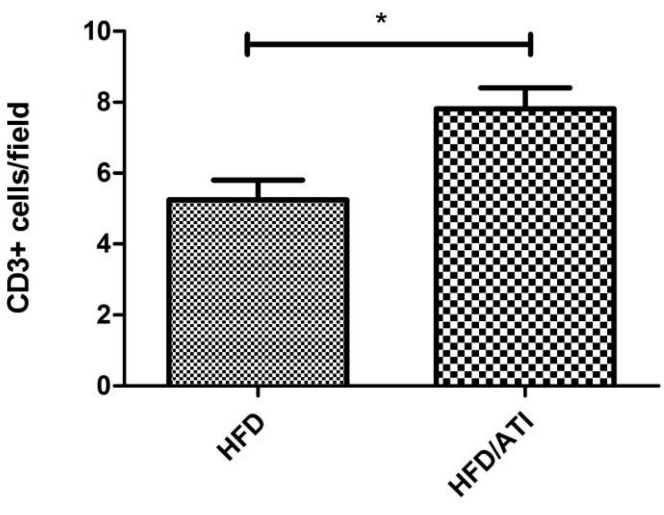

Suppl. Fig 5

A

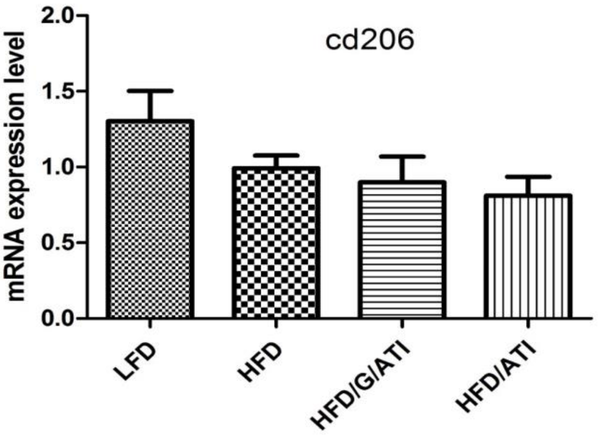

B

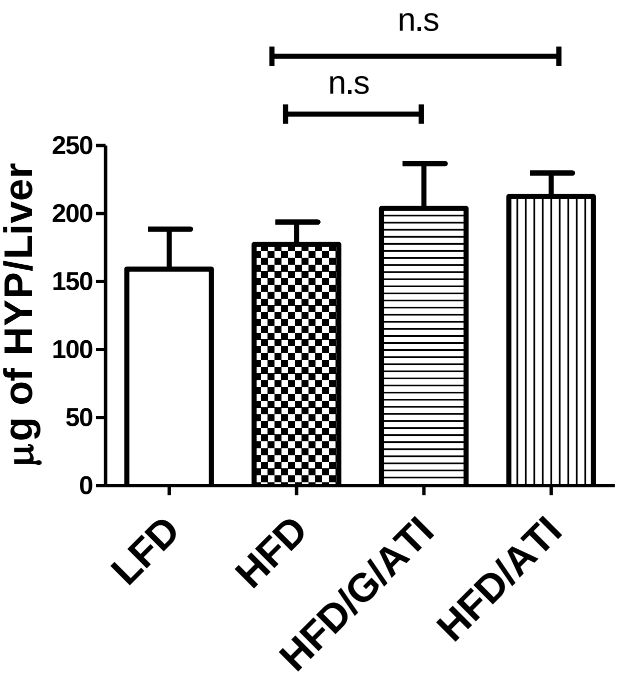

C

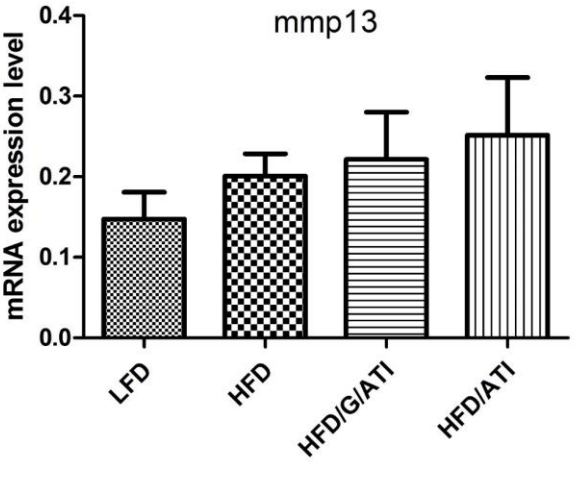

Diet Formula

Ingredients	Low fat zein Diet	High fat zein based diet	High fat with 30% wheat gluten zein based diet	High fat with 0.15% Purified ATI zein based diet
Zein (%)	22.100	22.100	15.470	21.6
Wheat gluten (%)	----	----	6.630	---
Purified wheat ATI				0.7
Corn starch (pre-gelatinzied) (%)	50.000	13.600	14.200	13.600
Maltodextrin (%)	5.000	5.000	5.000	5.000
Sucrose (%)	5.000	12.00	12.00	12.00
Cellulose (%)	5.200	7.700	7.700	7.700
DL-Methionine (%)	0.100	0.100	0.100	0.100
L-Cysteine (%)	0.200	0.200	0.100	0.200
Vitamin Premix (%)	1.000	1.000	1.000	1.000
Mineral & trace elements premix (%)	6.000	6.000	6.000	6.000
Choline chloride (%)	0.200	0.200	0.200	0.200
L-Lysine HCl (%)	1.800	1.800	1.800	1.800
L-Threonine (%)	0.300	0.300	0.180	0.300
L-Tryptophan (%)	0.230	0.230	0.180	0.230
L-Valine (%)	0.970	0.970	0.800	0.970
L-Isoleucine	0.300	0.300	0.250	0.300
L-Arginine, free base (%)	0.300	0.300	0.210	0.300
L-Histidine, free base (%)	0.100	0.100	0.050	0.100
Cholesterol (%)	0.100	0.100	0.100	0.100
Soybean oil (%)	5.300	0	0	0
Corn oil (%)		28.000	28.000	28.000

Suppl. Table: 1

Target gene	Forward primer (5’-3’)	Tagman	Reverse primer (5’-3’)
tgfß1	AGAGGTCACCCGCGTGC	ACCGCAACAACGCCATCT	TCCCGAATGTCTGACGT
mmp9	CAGGATAAACTGTATGGCTTCTGC	CTACCCGAGTGGACGCGACCGT	GCCGAGTTGCCCCCA
mmp2	CCGAGGACTATGACCGGGATAA	TCTGCCCCGAGACCGCTATGTCCA	CTTGTTGCCCAGGAAAGTGAAG
tnfα	CTCAGCCTCTTCTCATTC	CACCACGCTCTTCTGTCTACTGA	GCCATAGAACTGATGAGA
Arg1	GGTCCAGAAGAATGGAAGAGTCAG		CAGATATGCAGGGAGTCACC
il6	ACCAGAGGAAATTTTCAATAGGC		TGATGCACTTGCAGAAAACA
cd68	CTTCCCACAGGCAGCACAGAG		AATGATGAGAGGCAGCAAGAG
ym1	GGGCATACCTTTATCCTGAG		CCACTGAAGTCATCCATGTC
gapdh	AGGTCGGTGTGAACGGATTTG		GGGGTCGTTGATGGCAACA
timp1	TCCTCTTGTTGCTATCACTGATAGCTT	TTCTGCAACTCGGACCTGGTCATAAGG	CGCTGGTATAAGGTGGTCTCGTT
col1a1	TCCGGCTCCTGCTCCTCTTA	TTCTTGGCCATGCGTCAGGAGGG	GTATGCAGCTGACTTCAGGGATGT
Gapdh	GACGGCCGCATCTTCTTGT	CAGTGCCAGCCTCGTCCCGTAGA	CACACCGACCTTCACCATTTT
srebp1c	ACGGAGCCATGGATTGCACA		AAGGGTGCAGGTGTCACCTT
Fas	TGCTCCCAGCTGCAGGC		GCCCGGTAGCTCTGGGTGTA
Acc	ATGGGCGGAATGGTCTCTTTC		TGGGGACCTTGTCTTCATCAT

Suppl. Table 2 Primer list
